# Titanium Dioxide as a Catalyst Support in Heterogeneous Catalysis

**DOI:** 10.1155/2014/727496

**Published:** 2014-10-14

**Authors:** Samira Bagheri, Nurhidayatullaili Muhd Julkapli, Sharifah Bee Abd Hamid

**Affiliations:** Nanotechnology & Catalysis Research Centre (NANOCAT), IPS Building, University of Malaya, 50603 Kuala Lumpur, Malaysia

## Abstract

The lack of stability is a challenge for most heterogeneous catalysts. During operations, the agglomeration of particles may block the active sites of the catalyst, which is believed to contribute to its instability. Recently, titanium oxide (TiO_2_) was introduced as an alternative support material for heterogeneous catalyst due to the effect of its high surface area stabilizing the catalysts in its mesoporous structure. TiO_2_ supported metal catalysts have attracted interest due to TiO_2_ nanoparticles high activity for various reduction and oxidation reactions at low pressures and temperatures. Furthermore, TiO_2_ was found to be a good metal oxide catalyst support due to the strong metal support interaction, chemical stability, and acid-base property. The aforementioned properties make heterogeneous TiO_2_ supported catalysts show a high potential in photocatalyst-related applications, electrodes for wet solar cells, synthesis of fine chemicals, and others. This review focuses on TiO_2_ as a support material for heterogeneous catalysts and its potential applications.

## 1. Introduction

### 1.1. Essential Principle of Catalyst

The catalysis industry is a billion-dollar industry that accounts for the manufacture of 60% of all the chemicals that are utilized for most chemical processes [1–6]. Some of the products derived from catalytic processes include polymers [2], plastics [3], pharmaceuticals [4], and detergents [5]. After decades of research, systematic information on the catalytic properties of many catalysts has been established and accumulated. From the literature, it is concluded that one of the major limitations of the catalytic reaction is separation and distribution [7–9]. Many of real catalysts are made up of small (in the nanometer range) sizes. This consequently brought about the uncertainly and nonuniformity of the materials involved, the preparation methods, and surface conditions [8]. Heterogeneous catalyst was introduced to overcome the separation and distribution problems.

### 1.2. Importance of Heterogeneous Catalyst

Heterogeneous catalysts have become a crucial part of many industrial activities, such as organic synthesis, oil refining, and pollution control [10–15]. Modern heterogeneous catalysts consist of several elements in precise proportions [12]. Currently, heterogeneous catalysts are optimized for the greatest reaction rate, which in turn results in optimal selectivity [11–13]. It is possible to improve the heterogeneous catalyst activity over modifying the support by approaches such as nanotechnology and nanoscience or controlling the pore structure [14–16]. For heterogeneous catalysis, the problem of catalyst separation and recovery from the reaction matrix are addressed by using various catalyst supports to immobilize the particle [15]. This in turn provides a large enough surface area for the heterogeneous catalyst for it not to dissolve into the solution matrix [16]. Therefore, the heterogeneous catalyst with broad supports such as Al_2_O_3_, TiO_2_, ZrO_2_, ZnO, and others is applied based on its broad availability and cost-effective modes of synthesis.

### 1.3. Importance of Heterogeneous Catalyst Support

Recently, the importance of an appropriate catalyst's support material has been of huge interest. The idea is that the main catalyst should be dispersed on a suitable support to make the catalytic nanoparticles stable and obtain optimal performance and decrease the amount of costly metal being utilized, which accordingly decrease the total catalyst expenses [11, 15]. Furthermore, with porous characteristics, support materials offer a high dispersion of nanoparticle catalyst and simplify electron transfer, both of which contribute to better catalytic activities [17–20].

However, the heterogeneous catalyst support may sometime exert a structural effect, brought about by textural and active phase-linked effect [18]. Thus, the selection on support heterogeneous catalyst must retain its specific properties, such as porosity, surface area, dispersion, selectivity, and activity [19–21]. The morphology and pores size of the selected support materials play an important role in enhancing the heterogeneous catalyst's stability and performance [20].

According to the literature, the support of the heterogeneous catalyst can be alumina [22], zeolites [23], carbon nanofibers [19], active carbon [17], and metal oxides [13], such as TiO_2_, La_2_O_3_, CeO_2_, MnO_2_, and ZrO_2_. TiO_2_ is a recognized heterogeneous catalyst support that is broadly utilized in fuel processing due to its tunable porous surface and distribution, high thermal stability, and mechanical strength [24, 25]. Being used in this manner contributes to the ability of TiO_2_ to develop Lewis acidity as well as redox properties [25].

## 2. TiO_2_: In General

TiO_2_ has proven to be one of the promising* n*-type semiconductors due to its wide band gap (3.2 eV) under ultraviolet light [24]. Additionally, possessing high physical and chemical stability as well as the high refractive index makes this material widely researched [25]. Due to its electronic and optical properties, it can be utilized in several fields, such as solar cells, photocatalyst, sensors, and self-cleaning [26]. In electrochemistry, TiO_2_ based materials play a key role due to their high conductivity and stability in alkaline and acid media. TiO_2_ exists in three crystalline forms; anatase and rutile are the most common types, and the crystalline size of the rutile is always larger than the anatase phase. Brookite is the third structural form, an orthorhombic structure, which is rarely utilized, and is of no interest for most applications [27–30]. Rutile phase is the most thermally stable among the three phases. Brookite and anatase crystalline, above 600°C, experience a phase transition and convert into the rutile phase [28, 29]. The anatase phase contains zigzag chains of octahedral molecules linked to each other, while the rutile consists of linear chains of opposite edge-shared octahedral structure [29–32]. Generally, the anatase-to-rutile phase transformation occurs between 600–700°C, but, for certain applications, it is required that TiO_2_ anatase be stable at 900°C [31]. Generally, the anatase TiO_2_ nanoparticles are stabilized by the addition of cations [32].

The synthesis techniques of TiO_2_ usually require high temperatures to crystallize the amorphous material into one of the phases of TiO_2_, such as brookite, anatase, and rutile, consequently leading to larger particles and typically nonporous materials [33–35]. Recently, low temperature synthesis methods resulted in crystalline TiO_2_ with a higher degree of control over the formed polymorph and its intra- or interparticle porosities [32]. There are reports on the formation of crystalline nanoscale TiO_2_ particle via solution based approach without thermal treatment with special focus on the resulting polymorphs, surface area, particle dimensions, and crystal morphology [34]. There are exceptional emphases on the sol-gel method via glycosylated precursor and also the miniemulsion method [30–32].

TiO_2_, due to its nontoxicity, long-term photo stability, and high effectiveness, has been widely utilized in mineralizing toxic and nonbiodegradable environmental contaminants. TiO_2_ possesses good mechanical resistance and stabilities in acidic and oxidative environments. These properties make TiO_2_ a prime candidate for heterogeneous catalyst support.

### 2.1. TiO_2_: As a Heterogeneous Catalysis

It has been demonstrated that TiO_2_ improve the performance of catalysts [35–39], allowing the modulation of catalytic activities for many reactions, including dehydrogenation [38, 39], hydrodesulphurization [37], water gas shift [36], and thermal catalytic decomposition [35].

There are also some obvious drawbacks in using TiO_2_ as a heterogeneous catalysis. The limitations included small specific surface areas, low quantum efficiency, and low adsorption abilities [36, 37]. Furthermore, both costs and difficulties in the separation of catalyst from the reaction media and inadequacy for continuous processing limited the applications of TiO_2_ as a heterogeneous catalyst in large-scale industries [38]. Despite these drawbacks, a number of studies have focused on catalytic reaction with TiO_2_ as a support material. The mesoporous TiO_2_ of pure anatase phase with sharp pore distribution and large surface area was synthesized to increase the degree of distribution and homogeneity of immobilized catalyst [39, 40]. The influences of TiO_2_ support on heterogeneous catalysts affect electronic effects and bifunctional mechanism [41]. TiO_2_, as a catalyst support, enforces an electronic effect where the hypo-d-electronic Ti^3+^ promotes electrocatalytic features of hyper-d-electronic noble catalyst surface atoms [42]. This, in turn, decreases the adsorption energy of CO intermediates, while enhancing the mobility of CO groups. At the same time, the adsorption of OH species on TiO_2_ tends to facilitate the conversion of the catalytically toxic CO intermediates in CO_2_, thus improving the durability of the heterogeneous catalyst [43, 44]. Both factors indirectly assist the dispersion and anchor of the heterogeneous catalyst particle [44]. Further improvement in the catalytic stability and activity of the heterogeneous catalysts involves modifying the TiO_2_ support material with semiconductor metal oxides.

## 3. TiO_2_: As Support in Heterogeneous Catalysis

Among different material candidates such as nitrides, perovskites, and carbides, TiO_2_ based catalyst support materials are known to have excellent properties [44], due to TiO_2_ nanoparticles high chemical and thermal stability. TiO_2_ based catalyst supports have outstanding resistance towards corrosion in different electrolytic media. TiO_2_ can be regarded as a support for heterogeneous catalysts which guarantees stability in electrochemical environment and commercial availability [45]. Meanwhile, strong interactions between the catalytic particles and mesoporous TiO_2_ have been recorded, which, in the end, resulted in both improved catalytic stability and activity. TiO_2_ as a catalyst support material also indicated a certain degree of proton conductivity, which may potentially enhance the regime of the triple phase boundary for catalytic reactions [44–46]. In general, the advantages and disadvantages of the other heterogeneous catalyst system are listed in Table 1.

### 3.1. TiO_2_: As Support in Metal Heterogeneous Catalysis

The study of metal nanoparticle on TiO_2_ support is important in heterogeneous catalysis due to the size and nature of the interaction of a metal nanoparticle with TiO_2_ support [45]. This interaction strongly influences the determination of catalytic activity and selectivity of the metal heterogeneous catalyst [46]. Reduction and oxidation at elevated temperature are compulsory steps in the preparation of metal supported TiO_2_ heterogeneous catalysts [47, 48]. However, both treatments caused morphological changes to the dispersed metal nanoparticles from the sintering of TiO_2_. Therefore, it is important that the optimal conditions for catalyst supported TiO_2_ preparation be optimized, both in terms of pretreatment and activation [48, 49]. Besides, depending on the particular metal heterogeneous catalyst, different morphological changes will result from metal-TiO_2_ support interaction [50–52], such as sintering [50], alloy formation [52] encapsulation, and interdiffusion [51].

Among the TiO_2_ modifications, anatase is frequently utilized as a catalyst support for metal heterogeneous catalyst due to its high specific surface area and strong interaction with metal nanoparticles [37, 40]. There are only a few studies reporting a rutile catalyst support which resulted in higher catalytic activity compared to anatase, such as the oxidation of toluene, xylene, and benzene over rutile-supported Cu catalyst. In comparison, rutile is preferred as a model support for particles of metals in surface science studies [53–55], due to its high crystal phase's thermodynamic stability. Furthermore, it is indicated that rutile and anatase differ noticeably in their ability of fixing particles of metals onto their respective surface [49, 55]; whereas the strong metal support interaction is normally shown on anatase, this effect is not as significant on rutile. Inopportunely, the thermodynamic stability of TiO_2_ is comparatively low, and calcination would usually lead to the collapse of the porous structures [54]. Additionally, it is reported that calcination above 465°C has always resulted in the phase transition from anatase to rutile [36]. The phase transition could be connected to the growth of crystal size, which results in a severe reduction in specific surface area [35]. Consequently, this should also influence the overall catalytic performance of metal heterogeneous catalysts.

#### 3.1.1. Au/TiO_2_ Heterogeneous Catalyst

Gold (Au) is an excellent catalyst for the oxidation of alcohol by molecular O_2_ in the liquid phase with high activity, selectivity, and promising resistance to deactivation [56–58]. The catalytic performance of Au heterogeneous catalyst is mainly determined via the particle size and properties of the support [57]. Amongst all catalyst supports, TiO_2_ was determined to be a good support for the Au heterogeneous catalyst system due to the strong interaction in metal support, chemical stability, and acid-base properties [44, 46].

**Figure 1:**
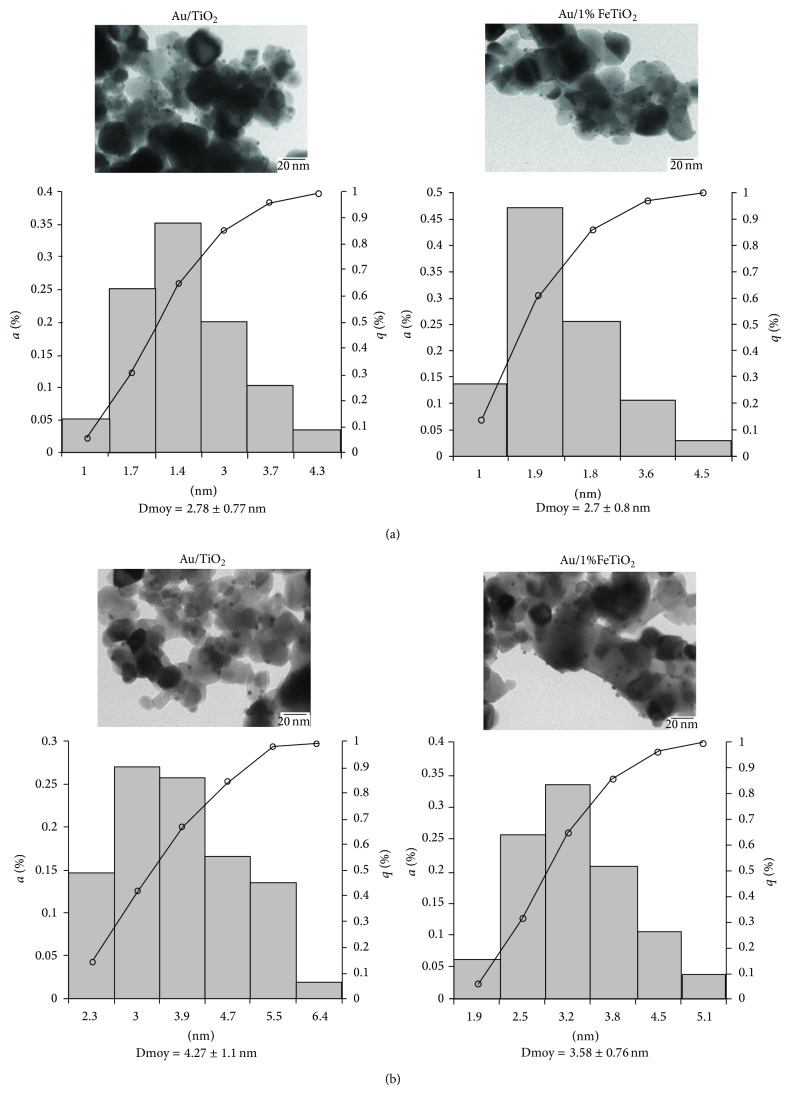
TEM analysis of gold supported on TiO_2_ and 1% FeTiO_2_ before (a) and after (b) thermal treatment [59].

Au-supported TiO_2_ nanoparticle has been prepared by changing the several different synthesis parameters, including precipitation pH, drying pretreatment, Au-cluster size/morphology, catalyst conditions, the nature of the support, acid-base treatment of TiO_2_ support, loading of TiO_2_ nanoparticle, incorporation of impurities, CO adsorption, catalytic reaction conditions, and chemical/electronic state in catalysts [53, 58]. The valence band of Au/TiO_2_ indicates the presence of the Au 5d band, with a significant contribution to the O 2p nonbonding states, which probably is derived from TiO_2_ structure [46]. Therefore, the morphology images showed an interaction among the particles of Au with TiO_2_ support affecting the Ti–O bonds at the surface, which leads to a lower Ti 2p binding energy in an intact Au/TiO_2_ compared to native TiO_2_ (Figure 1) [59–62]. Generally, Au nanoparticles, as a catalyst, have a negative charge [57]. This is due to the electron transfer from oxygen vacancies of the TiO_2_ acting as a catalyst support [58].

**Figure 2:**
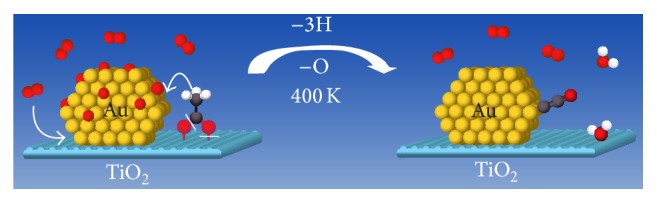
This shows the conversion of acetate to ketenylidene at the perimeter site of the Au/TiO_2_ catalyst. The acetate, which adsorbs on TiO_2_, undergoes dehydrogenation (oxidation) and the deoxygenation to form ketenylidene on the gold [60].

The transformation was clearly observed in the case of acetate to ketenylidene reaction [60] (Figure 2). Thus, the charge density and level binding energy of Au is 0.15–0.45 eV lower than that in pure Au [56]. It is concluded that the presence of TiO_2_ as a support in Au is necessary for the crystallization of the support. This, in turn, reduces the number of Ti–OH functions to be proportional to the deposition of Au [60].

The aforementioned theory brought about the next advantage of Au/TiO_2_ hybrid catalyst. The incorporation of Au is believed to prevent radiation-induced changes in the Au/TiO_2_ heterogeneous catalyst composition, particularly for as—synthesized and dried samples [63, 64]. For example, X-ray irradiation limits a detectable further reduction in the Au/TiO_2_ due to the presence of Au^3+^ state and binding energy of the Au itself [57]. The binding energy and half-width of the Au 4f_7/2_ spectra of Au particles deposited on the TiO_2_ in a range of concentrations were sensitive to irradiation. Indeed, the local heating mechanism occurred at higher X-ray concentrations [65]. Exposure of Au/TiO_2_ heterogeneous catalyst to X-ray irradiation induces the breaking of Ti–O bonds, Ti^4+^ state reduction, and the O_2_ desorption from the surface layer [64–66]. This is demonstrated by an increase in the fraction of Ti^3+^ species, a nonmonotonic diminution of the O_2_^−^/Ti^4+^ atomic ratio, improvement of the valence band, and the resultant variation of core binding energy [65]. Even though Ti 2p and O 1s binding energies in TiO_2_ remain almost unaffected under extended X-ray irradiation, in Au/TiO_2_, a gradual increase in the Ti 2p binding energy is observed [67]. Thus, the effect of X-ray irradiation of Au/TiO_2_ is relatively smaller than Au due to the direct evidence for charge transfer processes triggered by the production of O_2_ vacancies in TiO_2_ as a catalyst support [63, 64].

**Figure 3:**
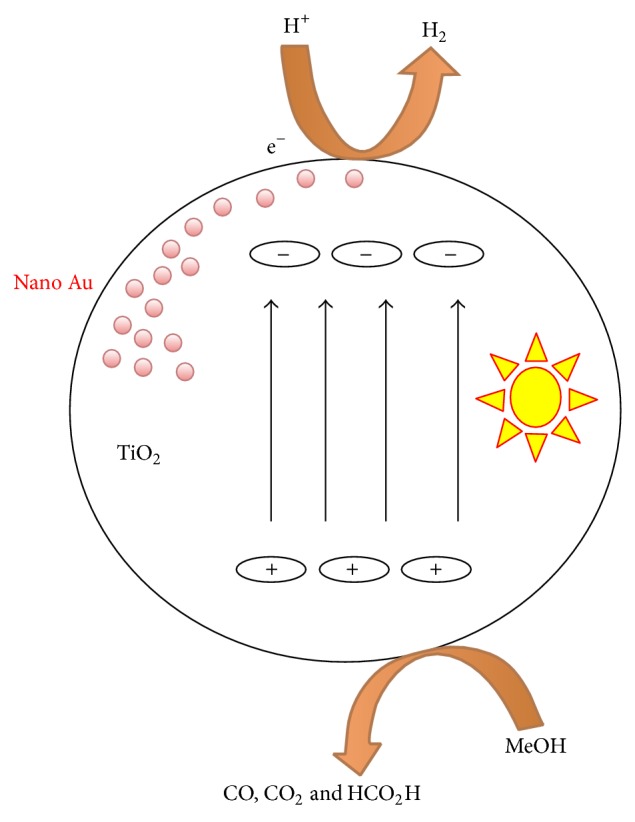
Au/TiO_2_ catalysts, easily prepared in situ from different Au precursors and TiO_2_, generate H_2_ from H_2_O/alcohol mixtures [73].

Many scientists have documented that Au/TiO_2_, as a heterogeneous catalyst, showed higher reaction activities for the oxidation of primary alcohols to carboxylic acid compared to Au/zeolite [68]. The comparative study of CO oxidation reaction and deactivation behavior between the mesoporous Au/TiO_2_ and Au demonstrated that Au/TiO_2_ has greater catalytic selectivity and activity due to its higher surface area. Another study found that the deposition of Au into TiO_2_ led to the highly active heterogeneous catalyst for the oxidation of methanol [69–71]. The catalytic performance was determined to increase with Au loading, provided that the deposited particle size remained unchanged [70–72]. In this case, the methoxy species bound to the oxide surface are reasonable reaction intermediates, with the formation of significant bound on TiO_2_ [71]. The catalytic performance was correlated with the number of Au atoms. Thus, the methanol oxidation occurs at the interface with O_2_ being activated at Au nanoparticle and the oxide acting as a reservoir of methoxy species (Figure 3) [73–75].

Furthermore, there are several reports on the catalytic application of Au/TiO_2_ on the removal of CO and NO_*x*_. Most of these reports investigated the catalytic and photocatalytic of Au/TiO_2_ in the gas and liquid phase reactions, as well as their reaction mechanisms [74]. In other studies a higher stability was observed for Au/TiO_2_ heterogeneous catalyst compared to Au/Co_3_O_4_, which indicates a comparatively fast deactivation [70, 72]. This is supported by the aforementioned report; a significant enhancement in the stability of Au particles against calcination can be realized by utilizing TiO_2_ as a catalyst support [71]. With these promising properties, Au/TiO_2_ catalyst was also found to catalyze the epoxidation of propylene, hydrogenation reaction, water-gas shift reaction, and the oxidation of alkanes [68–76].

Meanwhile, the porosity and phase transformation are other factors that affect the catalytic performance of Au/TiO_2_ as a heterogeneous catalyst [77, 78]. It is estimated that various TiO_2_ crystalline phases, as a catalyst support, could affect the interaction of support-metal, electronic density, oxidation state, Au-size, and Au dispersion of deposit material in heterogeneous catalyst system. In a comparative study of the activity of porous and nonporous Au/TiO_2_, heterogeneous catalyst prepared from different crystalline phase of TiO_2_ included the anatase, rutile, and rutile+anatase [69, 79]. For example, certain studies found that the activity of nonporous Au/TiO_2_ heterogeneous catalyst decreased from brookite via anatase to rutile for catalyst activated by calcination at 300°C, while, after reduction at 150°C, the activities became rather comparable [80, 81]. Similar observation was reported by other studies for mesoporous TiO_2_ acting as support for Au heterogeneous catalyst [82]. The tendency in the increment for Au particle sintering during calcination at 300°C is brookite, anatase, and rutile, followed by mesoporous. In contrast, such effect is insignificant at the reduction of 150°C [82]. Some studies reported that the crystalline phase of TiO_2_ influences also the deactivation of Au/TiO_2_ heterogeneous catalyst with comparable surface area of 46–54 m^2 ^g^−1^, increased in the order of Au/TiO_2_ anatase and Au/TiO_2_ nonporous rutile/anatase and followed by Au/TiO_2_ rutile [83]. Contrarily, some studies concluded that the crystalline phase has no significant influence on the catalytic performance of the unconditioned Au/TiO_2_ heterogeneous catalyst [84]. This was different, as the Au/TiO_2_ heterogeneous catalyst was calcined prior to the reaction, where the Au/TiO_2_ rutile+anatase heterogeneous catalyst indicated a significantly higher stability than Au/TiO_2_ rutile and Au/TiO_2_ anatase [85]. After calcination at 250°C, the activities are comparable to those of the unconditioned heterogeneous catalyst and are similar for all catalysts. However, calcination at 350°C leads to lower activities for the mesoporous rutile or anatase, while the rutile+anatase supported Au retains its respective activities [83]. The catalytic performance of Au-supported TiO_2_ anatase being superior to other heterogeneous catalyst was examined for the decrease of NO_*x*_ using propane due to the smaller size of the Au particles on TiO_2_ anatase [86, 87].

Although Au/TiO_2_ is active as a heterogeneous catalyst, it often complied with quick deactivate process which limits its commercial applications [70–74]. In general, in order for the Au particle to indicate higher performance at near subambient temperatures, its cluster size has to be less than 5 nm [65]. Therefore, some attempt has to be made to synthesize Au hydroxide [AuO_*x*_(OH)_4−2*x*_]_*n*_ deposited on the TiO_2_ support, followed by drying in the air. The idea is to prevent the in situ formation and the agglomeration of metallic Au nanoparticles, as seen with coprecipitation [64]. Another approach is by synthesizing nearly monodispersed thiol-protected Au nanoparticles and is deposited into TiO_2_ as a support. In this case, the thiol ligands were utilized to control the size of the cluster [82, 88]. The advantages offered by both methods included a better control size of Au nanoparticle due to* ex situ* synthesis and the formation of Au cluster in the solution prior to the deposition on the TiO_2_ as a catalyst support [88].

In conclusion, the most common factors affecting the Au/TiO_2_ heterogeneous catalyst activity are the size of the Au nanoparticles, preparation method, pretreatment method, Au loading, the oxidation state of Au, and the binding strength to TiO_2_ as a catalyst support.

#### 3.1.2. Co/TiO_2_ Heterogeneous Catalyst

**Figure 4:**
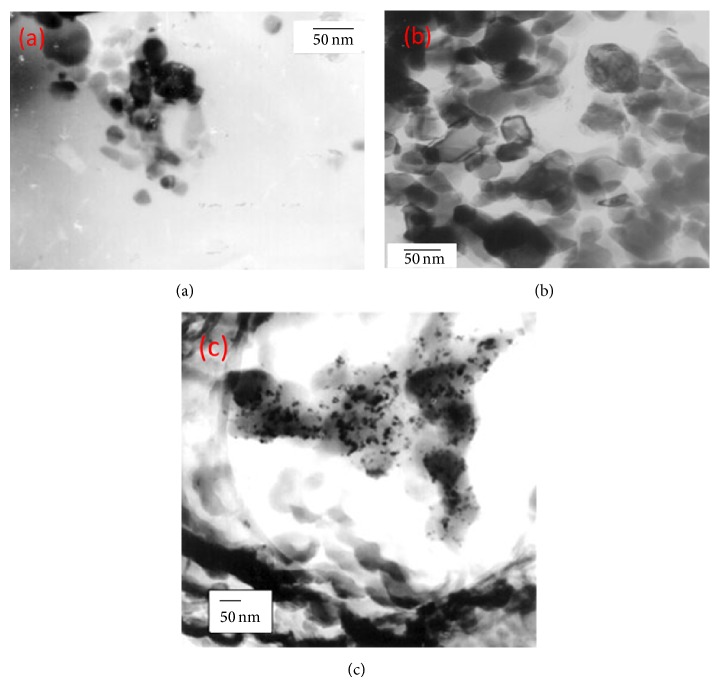
(a) TEM micrograph of Co/TiO_2_ (673)-I catalyst after reduction-oxidation-reduction cycle at 773-623-623 K. (b) TEM micrograph of Co/TiO_2_ (973)-I catalyst after reduction-oxidation-reduction cycle at 773-623-773. (c) TEM micrograph of Co/TiO_2_ (673)-SG catalyst after reduction-oxidation-reduction cycle at 773-623-773 K [97].

The activity of cobalt supported TiO_2_ (Co/TiO_2_) heterogeneous catalyst is greatly related to the TiO_2_ crystal phase and the loading of Co^3+^ ions on the catalyst support [89–91]. The case of Co/TiO_2_, TiO_2_ in the rutile phase is more appropriate as a catalyst support material than those with a 100% anatase phase [25, 90]. Therefore, Co/TiO_2_ heterogeneous catalyst synthesized with more than 15% rutile phase is shown to have 4 times higher CO conversion rate than those that only consist of the anatase phase [92, 93]. Furthermore, the Co/TiO_2_, when modified with alkaline earth metals, resulted in a greater CO conversion rate, whereas modification with Mn and V resulted in high C^5+^ and low methane selectivity, respectively [94–96]. Furthermore, with only 0.8% Ca modification, Co/TiO_2_ obtained the highest CO conversion rate and site-time yield of C^5+^ products. The conversion rate is 1.3–1.5 times higher than cosupported SiO_2_ catalyst (Figure 4) [97]. Therefore, the CO conversion reaction rate over Co/TiO_2_ catalyst was proportionately amplified by increasing the surface area of Co [93].

Actually, the production of highly dispersed Co on TiO_2_ as a catalyst support requires a strong interaction [91, 92]. Nevertheless, too strong of an interaction generates the TiO_2_ compound as a suboxide at an interface, which is highly resistant to reduction [89]. In this case, while it has known that the dominant surface sites of TiO_2_ support consist of two main sites, which are Ti^3+^ and Ti^4+^, the surface site's effect on the formation of Co to TiO_2_ interface indicates that the reaction should be structured to be insensitive and based on the number of the exposed Co metal sites [93–99]. It is also proposed that this growth in the reaction rate might be due to the strong Co to TiO_2_ interaction altering the catalytic properties [100].

#### 3.1.3. Ni/TiO_2_ Heterogeneous Catalyst

Nickel (Ni) supported TiO_2_ (Ni/TiO_2_), as a heterogeneous catalyst, is another kind of important Ni-based catalyst. This is due to the strong interaction between Ni as a metal and TiO_2_ as a catalyst support [101–103]. For example, the catalytic properties of supported Ni/TiO_2_ heterogeneous catalysts prepared by the incipient wetness impregnation method were evaluated for the vapor phase hydrogenation of maleic anhydride [104]. It was discovered that the hydrogenation process of maleic anhydride is strongly affected by the calcination temperature of Ni/TiO_2_. The Ni/TiO_2_ heterogeneous samples recorded an optimum catalytic performance with 96% maleic anhydride conversion as the calcination temperature reached 1023 K [104, 105]. This is attributed to the change of surface properties of TiO_2_ support to the increase of calcination temperature. In addition, the strong metal support interaction between Ni and rutile surface over Ni/TiO_2_ catalyst was the key reason for the better activity of the catalyst in acetophenone hydrogenation [105]. However, the deactivation of Ni/TiO_2_ heterogeneous catalyst occurred as the carbonaceous species of maleic anhydride was deposited onto the Ni surface [106, 107]. To regenerate the catalytic performance of Ni/TiO_2_, thermal treatment in the oxidant atmosphere approach is applied.

#### 3.1.4. Pd/TiO_2_ Heterogeneous Catalyst

Anatase TiO_2_ effectively engenders OH species. Thus, palladium-supported TiO_2_ (Pd/TiO_2_) anatase possesses excellent catalytic activity vis-à-vis methanol electrooxidation. There are some reports claiming that Pd-supported TiO_2_ anatase heterogeneous catalyst demonstrated a greater activity than TiO_2_ rutile in selective hydrogenation reactions due to the superior metal supporting the behavior of TiO_2_ anatase [108–112]. Consequently, negligible CO intermediates are produced in direct formic acid fuel cells and formic acid electrooxidation, which consequently possess striking catalytic activity. In contradiction, the acetoxylation of toluene study indicates that Pd supported by rutile TiO_2_ has greater selectivity of almost 100% without any deactivation of the catalyst compared to the ones supported by anatase [110–113], due to the high thermal stability of rutile. Meanwhile, the treatment of Pd/TiO_2_ anatase heterogeneous catalyst by an H_2_ reduction at 200°C resulted in greater reactivity in hydrogenation of alkadienes than that of the Pd/TiO_2_ in rutile phase [112]. The strong metal support interaction was only observed in the anatase phase of supported Pd heterogeneous catalyst [109, 110]. There is also an attempt to synthesize the genesis of the Pd cluster on TiO_2_-grafted SiO_2_. The results showed that TiO_2_ anchors Pd particles during air calcination and maintains its small ensemble during H_2_ reduction [114, 115]. These observations serve to demonstrate how TiO_2_, as a catalyst support, influences the structure and catalytic performance, specifically for sulfur-resistant catalysts [114]. It was also concluded that TiO_2_-grafted SiO_2_ offers better support due to the presence of high surface area and thermal stability for Pd heterogeneous catalyst compared to pure TiO_2_ [116–118].

#### 3.1.5. Pt/TiO_2_ Heterogeneous Catalyst

The dispersion and loading of platinum (Pt) nanoparticles onto TiO_2_ support is controlled by the structure and porosity of TiO_2_. Consequently, a suitable combination of passable electronic conductivity and nanostructured morphology with controlled porosity could result in very promising Pt/TiO_2_ heterogeneous catalysts [119–124]. For example, a novel electrocatalyst based on mesoporous TiO_2_ supported Pt nanoparticles indicates a high stability under accelerated stress test conditions and activity compared to commercial Pt-supported carbon catalyst [121, 122]. Some studies have investigated the influence of the reductive treatment on structural properties of Pt/TiO_2_, with their catalytic activity for formaldehyde oxidation [123]. It is claimed that the enhanced catalytic performance is reflected by a uniform dispersion of Pt nanoparticles and the interaction between Pt and TiO_2_ [124]. Thus, the application of Pt as a heterogeneous catalyst becomes more encouraging, especially in electrochemical and photoelectrochemical context [125, 126]. For example, H_2_ production could be significantly improved by photocatalytic water splitting over Pt/TiO_2_ nanosheets compared to native Pt [126].

**Figure 5:**
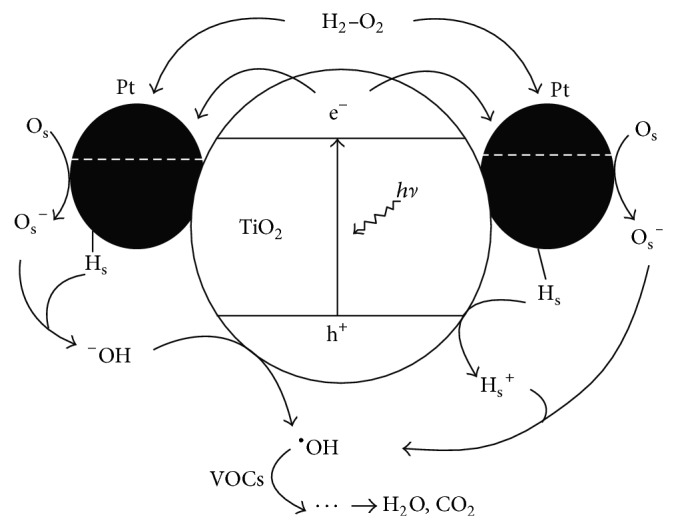
Proposed mechanism for the photochemical generation of dOH radicals on a Pt/TiO_2_ catalyst in the coexistence of H_2_ and O_2_ [136].

Several methods have applied in the preparation of Pt nanoparticle deposited on TiO_2_ substrates included underpotential deposition [127–129], hydrothermal treatment [130, 131], photoassisted reduction [132], and vacuum deposition [133, 134]. Electrodeposition would be a straightforward approach for the synthesis of Pt-supported TiO_2_, which is highly required for the production of complex electrode architectures in fuel cells [132–134]. This in turn resulted in the H_2_ evolution rate of 3 wt% Pt/TiO_2_, which was relatively greater than that of 3 wt% Pt/Al_2_O_3_. Other studies found that TiO_2_, as a support material, improves Pt O_2_ reduction activity by assisting mechanisms such as reactant surface diffusion and O_2_ spillover [135–137]. Furthermore, Pt/TiO_2_ showed similar performance to Pt/C in H_2_-fuel cell operated at 60°C and 0.8 V (Figure 5) [136].

Certain studies on the effect of particle size indicated that amorphous TiO_2_ could powerfully suppress the O_2_ reduction reaction activity of supported Pt at smaller sizes [138–140]. For example, a series of Pt/TiO_2_ heterogeneous catalyst with various Pt particle sizes was prepared and tested for low temperature CO oxidation [139]. The result indicated that the Pt/TiO_2_ heterogeneous catalyst that resulted in superior activity at the weight percent of Pt in Pt/TiO_2_ heterogeneous catalyst was 5.0%, with the complete conversion temperature being 120°C [141–143].

### 3.2. TiO_2_: As Support in Metal Oxide Heterogeneous Catalysis

Metal oxides signify one of the important and broadly used categories of heterogeneous catalysts. Metal oxides are utilized for both their redox and their acid-base properties and constitute the main family of catalyst in the heterogeneous category. Furthermore, certain metal salts and organometallic compounds using a heterogeneous catalyst precursor have the tendency to be decomposed via exposure to light irradiation. Some reports show that, as the oxide-containing catalyst under light irradiation analysis needs great care, considerable damage is possible even at low irradiation dose. For catalytic applications, metal oxides have also been employed for various applications such as gas sensor, the photocatalyst thin film, and fuel cells. TiO_2_ is often used to modify the supported metal oxide heterogeneous catalyst due to properties such as reducible surface and possible electron transfer via the spontaneous alignment of the Fermi levels.

#### 3.2.1. CuO/TiO_2_ Heterogeneous Catalyst

It has been reported that copper oxide (CuO) heterogeneous catalyst is highly active for CO oxidation and lower in cost compared to other noble metal heterogeneous catalysts [144–147]. CuO has additional advantages, such as a high thermal stability and the fact that it is economical [145–149]. The highest activities of CuO heterogeneous catalyst are attributed to the synergy between the Cu species and the support, especially in the case of TiO_2_ as support material. For example, the increment of activity for CO oxidation over the CuO/TiO_2_ heterogeneous catalyst is attributed to the sites for CO chemisorption that is responsible for O_2_ activation [150, 151]. Furthermore, since CO oxidation over the supported metal heterogeneous catalyst takes place at the metal support interface, TiO_2_, as reducible oxide, could provide the intrinsic activity to the entire reaction [149]. Therefore, only a small amount of CuO is loaded onto TiO_2_ and influence of TiO_2_ crystalline phase, which are indicative of the fact that the support system has higher efficiency and selectivity than the CO oxidation being examined [152].

On the other hand, CuO/TiO_2_ heterogeneous catalyst is also found suitable for NO reductions [150]. Rutile TiO_2_ is the most stable phase compared to anatase and brookite, and Cu-based heterogeneous catalysts show promising activity towards NO reduction [151]. Therefore, it is necessary and important to further approach the change of heterogeneous catalyst surface with plasma-assisted processing. NO reduction by CO reaction was comparatively studied over microwave plasma pretreated CuO/TiO_2_ heterogeneous catalysts employing transmission electron microscopy, H_2_ temperature programmed reduction, and in situ Fourier transform infrared spectroscopy [152–154]. The CuO/TiO_2_ heterogeneous catalytic performances indicated that a remarkable improvement in activity and selectivity was achieved after microwave plasma pretreatment, which depends on the microwave plasma pretreatment time [153]. The results also suggested that high active oxygen species are formed on the surface of plasma pretreated CuO/TiO_2_ heterogeneous catalysts, which led to the easy oxidation of NO to NO_2_ at low temperatures even without the introduction of any additional O_2_ gas. Therefore, these high active oxygen species should play an important role in the exaltation of the catalytic performances of the CuO/TiO_2_ heterogeneous catalysts [155]. It is found that CuO supported on the anatase phase of TiO_2_-support calcined at 400°C demonstrated better catalytic activity than those supported on TiO_2_ calcined at 500 or 700°C. Among all of the investigated heterogeneous catalysts with CuO loading from 2% to 12%, the CuO/TiO_2_ heterogeneous catalyst with 8 wt% CuO loading exhibited the highest catalytic activity [156]. The optimum calcination temperature of the CuO/TiO_2_ heterogeneous catalysts was recorded at 300°C.

CuO/TiO_2_ is also known as a promising heterogeneous catalyst in the photocatalytic applications. Whilst it is widely recognized that CuO facilitates charge separation and acts as a H_2_O reduction site, conjecture still exists as to the exact nature of the dispersed CuO, specifically on TiO_2_ and the optimum CuO loading for efficient H_2_ production [157, 158]. The surface chemistry of the CuO/TiO_2_ heterogeneous catalyst is the subject of a number of investigations to explain the excellent activity of the photocatalysts. It is generally recognized that CuO exists in several different forms on TiO_2_, with the specification depending on the CuO content and catalyst pretreatment conditions [159]. Experimental evidence suggested that, at low CuO loadings, Cu^2+^ is highly dispersed on TiO_2_ as a surface complex or CuO monolayer. This, in turn, can easily reduce the CO or H_2_ at low temperatures [150]. For example, CuO/TiO_2_ photocatalyst is very active in the reduction of water under sacrificial conditions (95 vol.% H_2_O, 5 vol.% CH_3_OH) and UV excitation, with the optimal CuO loading of 5 to 10 wt% of the heterogeneous catalyst system [153]. The high catalytic activity caused photo-citation of electrons in the conduction bands of both CuO and TiO_2_, followed by the migration of the conduction band electrons in TiO_2_ into the conduction band of CuO heterogeneous catalyst [155, 156]. The accumulation of excess electrons in the conduction band of CuO caused a negative shift in the Fermi level of CuO to give the required overvoltage necessary for H_2_O reduction [154]. However, once the CuO loading exceeds the dispersion capacity, nanocrystalline of the CuO begins to form. CuO is itself inactive for H_2_ production from water or alcohol-water mixtures under UV or visible irradiation, since the conduction band of CuO is more positive than the H_2_O/H_2_ redox potential [160–162]. Accordingly, the onset of the CuO nanoparticle formation should coincide with a decrease in the photocatalytic activity of CuO/TiO_2_ photocatalysts for H_2_ production, as the presence of inert CuO nanoparticles will reduce the number of surface sites on TiO_2_ available for photoreactions [161, 162]. Experimental evidence to support this hypothesis is currently lacking, which motivates the present investigation.

CuO/TiO_2_ heterogeneous catalyst could be synthesized by a deposition-precipitation method. For example, CuO has been deposited on TiO_2_ nanotubes with Cu/Ti atom ratio of 10 and then evaluated by H_2_ generation activity in methanol H_2_O mixtures under UV excitation. It is reported that the optimal CuO loading is around 1.3 wt%, giving the H_2_ production rate of 2061 *μ*mol g^−1 ^h^−1^[161]. Meanwhile, the CuO/TiO_2_ heterogeneous catalyst prepared by a complex precipitation method indicates the highest H_2_ production observed in 10 wt% of the CuO loading [162]. Another study evaluated the performance of 2.5 wt% CuO-TiO_2_ photocatalyst for H_2_ production from methanol and found that 27–29% of H_2_ evolution at rate of 1350 *μ*mol g^−1 ^h^−1^ is produced for ethanol-H_2_O mixture of 900–1000 g^−1 ^h^−1^ [158, 159]. It was recorded that 1 wt% CuO/TiO_2_ photocatalyst is active for H_2_O splitting under visible light in the presence of triethanolamine.

#### 3.2.2. V_2_O_5_/TiO_2_ Heterogeneous Catalyst

Initially, vanadium oxide (V_2_O_5_) supported TiO_2_ heterogeneous catalyst was applied for the o-xylene oxidation to phthalic anhydride reaction. Then, the potential of V_2_O_5_/TiO_2_ was recorded in the pollution abatement of NO with NH_3_ process [163, 164]. Thus, V_2_O_5_/TiO_2_ is one of the most efficient catalysts in the oxidative dehydrogenation of propane, with only a few byproducts [165]. A stable deposit of TiO_2_ in V_2_O_5_/TiO_2_ heterogeneous catalyst system was established with dip coating of V_2_O_5_ in an aqueous suspension of titanium isopropoxide [166, 167].

Furthermore, V_2_O_5_/TiO_2_ heterogeneous catalyst found another promising application in the dehydrogenation of alkanes and selective oxidation of alcohols/alkanes [168]. In this respect, the activity and product distribution of the heterogeneous catalyst for methanol oxidation strongly depend on its surface acidity and redox ability, and this can be regulated through proper modifications [169]. The heterogeneous catalyst of V_2_O_5_ supported on TiO_2_ exhibited high conversion of methanol and selectivity of dimethylmethane for the oxidation of methanol under mild conditions [170]. The catalytic oxidation of V_2_O_5_/TiO_2_ heterogeneous catalyst was investigated for 1,2-dichlorobenzene [171], and it was surmised that the catalytic oxidation of 1,2-dichlorobenzene on chemical vapor condensation which prepared V_2_O_5_/TiO_2_ heterogeneous catalyst demonstrated excellent performance at lower temperatures [171]. The surface structure of TiO_2_ allows the development of the Lewis acidity, as well as redox properties. Consequently, redox properties TiO_2_ modified by the presence of V_2_O_5_ lead to an electronic interaction between this support and V_2_O_5_ species [169]. V_2_O_5_ loading is a key point for the activity, and it has been suggested that a single redox surface site participates in the kinetically significant steps, with the formation of crystalline V_2_O_5_ being detrimental to oxidation activity [168, 172].

The effect on surface acidity and redox ability of V_2_O_5_/TiO_2_ is also found to be interesting for the selective catalytic reduction by NH_3_. The proposed mechanism suggested that NH_3_ is activated and reacts from a strongly adsorbed state with gaseous or weakly adsorbed NO [173–175]. It is believed that reliable structure-activity relationship is based on the understanding of the V_2_O_5_/TiO_2_ heterogeneous catalyst molecular structure under operating conditions [174]. Thus, a dual-site mechanism involving a surface V_2_O_5_ redox site and an adjacent nonreducible site appears to be more favorable. Furthermore, the reported influence of specific oxide supports, along with the observed stability of terminal V=^18^O bonds during NH_3_ reaction, suggests that the V–O-support bond is involved in the rate-determining step [167–169]. Similarly, the NH_3_ action of transition for V_2_O_5_/TiO_2_ heterogeneous catalyst correlates well with the extent of interactions between the active phase and the support [172]. The Raman studies of V_2_O_5_/TiO_2_ heterogeneous catalyst revealed that the presence of multiple structures of surface V_2_O_5_ species on TiO_2_, including monomeric and polymeric V_2_O_5_ species at submonolayer coverage [175, 176]. Therefore, Raman studies of V_2_O_5_/TiO_2_ heterogeneous catalysts under SCR or reducing (NH_3_) conditions are scarce.

The influence on preparation procedure towards surface of V_2_O_5_ state plays an important role in selective catalytic reduction. Some studies have investigated the surface of V_2_O_5_ prepared by incipient wetness impregnation, with TiO_2_ being a support, and found that both isolated and polymeric surface V_2_O_5_ species existed with medium VO surface coverage [177–179]. This is supported by other studies mentioning that polymeric V_2_O_5_ species demonstrated greater catalytic activity than monomeric V_2_O_5_ [178]. This is due to the greater mobility of lattice O_2_ atoms resultant experience faster reduction and reoxidation by gaseous O_2_ [179, 180]. Furthermore, the redox properties of V_2_O_5_ are modified by the incorporation of TiO_2_, leading to an electronic interaction between TiO_2_ and V_2_O_5_. There are some reports discussing the fact that the addition of molybdenum oxides (MoO_2_) species to V_2_O_5_/TiO_2_ heterogeneous catalyst causes dramatic changes in the 1,2-dichlorobenzene oxidation, most importantly, by improving the catalytic activity [181, 182].

#### 3.2.3. MnO/TiO_2_ Heterogeneous Catalyst

Manganese oxides (MnO), containing several types of labile oxygen, which are necessary to complete the catalytic cycle, have relatively high activity, but their optimal temperature (above 150°C) is still high [183–185]. For example, the activity and selectivity of pure MnO on NO conversion reached 90% selectivity [184]. To enhance the catalytic activity, MnO are usually supported on TiO_2_ as a carrier. In this regard, the dispersion of the MnO towards TiO_2_ support had an important influence on the reaction, since crystalline MnO contributed little to activity [186, 187]. This, in turn, possesses profound surface acid-base properties and provides high surface area, strong mechanical strength, and high thermal stability [188].

Existing research on supported MnO to TiO_2_ heterogeneous catalyst for NO oxidation demonstrated high catalytic activity [189–192]. For example, a series of MnO/TiO_2_ heterogeneous catalyst prepared by the deposition-precipitation method and the sample with the Mn/Ti ratio of 0.3 showed a superior activity for NO catalytic oxidation to NO_2_ [188]. Therefore, the maximum NO conversion over the MnO/TiO_2_ heterogeneous catalyst could reach 89% in 250°C [189]. Indeed, MnO/TiO_2_ heterogeneous catalyst showed an interesting development as a highly active heterogeneous catalyst with low pollution for the low temperature NO conversion process [190]. Meanwhile, some studies reported on MnO/TiO_2_ heterogeneous catalyst being prepared by sol-gel, impregnation, and coprecipitation methods for low-temperature selective catalytic reduction of NO with NH_3_ [191, 192]. Strong interaction, high concentration of hydroxyl groups, large surface area, and high concentration of amorphous Mn on the surface might be the main reasons for the excellent performance of the catalysts [191].

It was documented that the crystal phase of the TiO_2_ influences catalysts' activity [193–197]. Therefore, some studies on the effect of the crystalline phase of TiO_2_ towards the catalytic performance of MnO/TiO_2_ heterogeneous catalyst were carried out in [194], and it was discovered that compared to anatase and rutile anatase+rutile resulted in better dispersion of MnO on the support surface, suppressed the agglomeration of catalyst particles, and produced more Mn_2_O_3_, which is more active for the oxidation of NO [195, 196]. In addition, anatase+rutile enhanced the reduction of MnO, especially for Mn_2_O_3_, and the formation of easily desorbed O^2−^ generated from the Mn^3+^–O bond [197].

#### 3.2.4. RuO_2_/TiO_2_ Heterogeneous Catalyst

Ruthenium oxide supported TiO_2_ (RuO_2_/TiO_2_) heterogeneous catalyst found an attraction in the oxidation process [198, 199]. Generally, RuO_2_/TiO_2_ heterogeneous catalysts are prepared by the impregnation of Ru salt, followed by calcination, with limited control on the properties of the RuO_2_ species formation [199]. Furthermore, a recent development of green method to prepare calibrated RuO_2_ nanoparticles has been developed and analyzed. It is expected that, for this kind of preparation method, the RuO_2_/TiO_2_ heterogeneous catalyst exhibits outstanding oxidation activity [200].

### 3.3. TiO_2_: As Support in Bimetallic Heterogeneous Catalysis

In this area, the availability, affordability, and lack of toxicity of TiO_2_ as a robust solid with outstanding photochemical stability make it an attractive support for the bimetallic heterogeneous catalyst. TiO_2_ is used for its well-known ability to interact with bimetallic through the formation of Ti^3+^ ions. Generally, any electronic conductivity of TiO_2_ is due to the presence of Ti^3+^ ions. There are two ways to create Ti^3+^ ions in the TiO_2_ structure. The formation of Ti^3+^ ions either through the O_2_ vacancy creation or through shear planes by introducing appropriate donor dopants. It is well known that strong bimetallic support interaction occurred as the bimetallic catalyst was reduced by H_2_. The H_2_ reduction over TiO_2_ supported bimetallic catalyst generates O_2_ vacancies in the form of coordinate unsaturated cations in the vicinity of active bimetallic, which in the end results in changes in the catalytic activity and stability.

#### 3.3.1. PdNi/TiO_2_ Heterogeneous Catalyst

Spherical TiO_2_ nanoparticles were used to synthesize the PdNi-supported TiO_2_ electrocatalyst for methanol oxidation [201]. It was found that the electrocatalytic activity of PdNi/TiO_2_ catalyst is much more promising, better than the antipoisoning capability, and comparatively favorable as compared to commercial 31 PtRu-supported carbon [201]. The methanol oxidation mechanism of the PdNi/TiO_2_ heterogeneous catalyst mainly results from the high catalytic activity of the hybrid system without UV light illumination. Therefore, PdNi/TiO_2_ catalyst might become a promising candidate for a direct-methanol fuel cell.

#### 3.3.2. AuCu/TiO_2_ Heterogeneous Catalyst

The AuCu/TiO_2_ heterogeneous catalyst was studied for several other reactions, including water, gas shift, and total oxidation of methane, ethane, propane, and epoxidation of propane. The incorporation of Au into Cu complements each other in terms of electronic properties, O_2_ mobility, and surface stability [202, 203]. The interaction of Au and Cu in AuCu/TiO_2_ bimetallic heterogeneous catalyst was analyzed for methanol oxidation, and it was found that the catalytic activity and selectivity of the bimetallic heterogeneous catalyst system are greater than the one with the monolithic catalyst [202].

#### 3.3.3. CoMn/TiO_2_ Heterogeneous Catalyst

A series of CoMn/TiO_2_ heterogeneous catalysts, with a composition range of 2–12 wt% containing 25% Co and 75% Mn, have been prepared by the coimpregnation method [204–206]. The produced CoMn/TiO_2_ heterogeneous catalyst was tested in Fischer-Tropsch synthesis for the production of C2–C4 olefins [205], and it was discovered that the heterogeneous catalyst containing 8 wt% (CoMn)/TiO_2_ is the optimal formulation for the production of C2–C4 olefins. It should also be pointed out that the operating conditions, such as the H_2_/CO molar feed ratio, temperature, Gas Hourly Space Velocity, and total reaction pressure affect the heterogeneous catalytic performance of an optimal catalyst [204].

## 4. Applications of TiO_2_ Supported Heterogeneous Catalysis

### 4.1. Environmental Security: Photocatalysis

Recently, TiO_2_-supported semiconductor is extensively used to mineralize toxic and nonbiodegradable environmental pollutants due to its high effectiveness, long-term photostability, and nontoxicity [66, 76]. This is also attributed to the limitation of most semiconductors, such as low quantum efficiency, small specific surface area, and low adsorption ability. This in turn limits the efficiency of the photocatalyst. On the other hand, both costly and difficult separation of reaction media and the inadequacy for continuous processing are some of the restrictive factors [110]. As a result, a number of studies have focused on the immobilization of semiconductor materials onto porous TiO_2_ nanoparticles [156]. This is believed to not only promote photocatalytic reactions by offering more active sites but also allow the recycling and reuse of semiconductor as a heterogeneous catalyst. Some data also reported that porous TiO_2_ nanoparticle has shown some advantages in the preparation of highly supported catalysts due to its special physicochemical properties, including high adsorption capabilities [66].

Indeed, it is well known that TiO_2_, with its crystallographic forms, small particle size, and highly porous structure, greatly influence the photocatalytic performance of composite materials [76]. Among metal oxides suitable for photocatalytic processes, TiO_2_ is the most widely used, due to both its high photocatalytic activity and its chemical/photocorrosion stability in the reaction conditions. TiO_2_ has increased the photoactivities, due to the photoinduced electron-hole pairs on its surface that can be harvested to increase electron transfer and chemical reactivity. The semiconductor nature of TiO_2_ has made it possible for the utilization of UV-Visible radiation to harvest the conduction band electrons that are subsequently used to reduce metallic ions onto TiO_2_'s surface [66, 156]. Therefore, the immobilization of semiconductor on a TiO_2_ nanoparticle can exhibit a higher photodecomposition of organic and inorganic pollutant compared to nonsupported semiconductors [110].

### 4.2. Chemical Reaction/Conversion

As a versatile metal/metal oxide supported TiO_2_ heterogeneous catalyst, it is broadly studied in a variety of mild oxidation reactions, such as ethane to acetic acid, ethanol to acetaldehyde, and oxidative dehydrogenation of propane to propylene [8, 9, 16]. For example, various TiO_2_ supported catalysts, including Au/TiO_2_, Pd/TiO_2_, Co/TiO_2_, and Pt/TiO_2_, have recently been developed for frequent industrial applications, including the hydrosulfurization of hydrocarbon oils, the epoxidation of propane, and the selective catalytic reduction of NO [36, 43, 46–48]. However, since these aforementioned reactions are powerfully exothermic, it is important to avoid the hot spots that are responsible for structural damages and early deactivation of heterogeneous catalyst [53, 56–59]. Generally, the presence of hot spot leading from products productions included CO_2_, especially in oxidation reaction [60]. As an alternative, recent studies focused on the 3D structure of metal/metal oxide supported TiO_2_ with an open structure [63]. The open structure of metal/metal oxide supported TiO_2_ catalyst favor efficient heat and mass transfers between the gaseous reactants, the catalytic active phase, and the wall of the chemical reactors.

Furthermore, metal oxide supported TiO_2_ heterogeneous catalysts are commonly used in several industrial important reactions, including selective reduction of NO by NH_3_ [109, 121]. It should be pointed out that metal oxide supported TiO_2_ heterogeneous catalyst demonstrated excellent performance in slurry reactions [123, 128]. Indeed, TiO_2_, utilized as support for metal oxide, attracts considerable interest due to its favorable properties of low pressure drop, thermal shock resistance, chemical durability, low manufacturing cost, and high structural strengths [134–136, 160].

#### 4.2.1. Small Molecules Transformation

Recently, heterogeneous catalyst supported TiO_2_ for small molecules transformation has received considerable attention because of its simplicity and the advantages, over most other methods of preparing highly pure mixed oxides and a variety of other materials [13]. This included sulfides and phosphates, which are obtained under very similar experimental conditions. The preparation of metal particles with TiO_2_ supported catalyst is commonly applied because of its mild reducing performance which has a chelating effect, which avoids agglomeration of particles during preparation [57]. The synthesis of mono- or polymetal particles of Co, Ni, Cu, and noble metals in submicrometer and -nanometer size range has been reported and the materials obtained by catalyzed supported of TiO_2_ show homogenous phase composition, narrow particle distribution, and high specific surface area [203]. For example, polyol-mediated preparation of nanoscale oxides can carry out by dissolving a suitable metal precursor (acetate, alcoholate, and halogenide) in diethylene glycol or other polyalcohol with assisted of heterogeneous catalyst supported TiO_2_. During this step, the surface of growing particles will be immediately complexed by TiO_2_ as a catalyst support material, which limit grain growth [49].

**Figure 6:**
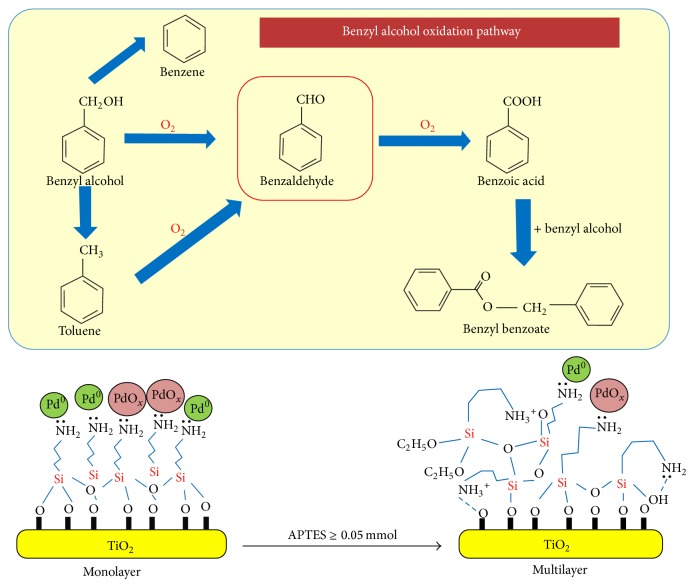
Surface functionalized TiO_2_ supported Pd catalysts for solvent-free selective oxidation of benzyl alcohol [207].

Heterogeneous catalyst supported TiO_2_ also applied for the nitrate to nitrite reduction with bimetallic catalyst. It is expected that H_2_ molecules produced through the reaction and adsorbed on noble metal subsequently reduces nitrite to harmless N_2_ gas [207]. A wide range of metal pairs included Au-Pd, Sn-Pd, Ni-Rh, Rh-Cu, and Pd-Cu supported on TiO_2_ were extensively studied to maximize the efficiency of nitrate reduction to N_2_ gas. In advances, some studies focus on the effect of pH and zwitterionic buffer on catalytic nitrate reduction by Cu-Pd supported TiO_2_ and found that the nitrate reduction decreased from 100% to 72% as suspension of pH increased from 6 to 10 of which range was kept by zwitterionic buffers [207] (Figure 6).

#### 4.2.2. Organic Synthesis

It also revealed that Pd catalyst supported TiO_2_ could be functionalized with various amounts of 3-aminopropyltriethoxysilane via a post synthesis grafting method combined with electroless deposition of Pd [108]. The supported catalyst system gave promising catalytic properties in the solvent-free selective oxidative of benzyl alcohol. It correlated well with the highest amount of Pd distribution (with particle size of 3.4 nm) with the 1% of Pd-supported TiO_2_. Increasing of surface basicity via the hydrolysis of NH_2_ suggested enhancing the dehydrogenation of benzyl alcohol and, as a consequence, the selectivity towards benzaldehyde [109–111]. In addition, the presence of TiO_2_ support gave high catalytic activity in benzyl alcohol oxidation, emphasizing that the reduction of PdOx species by the adsorbed benzyl alcohol is an essential step to form highly active metallic PdO sites [112].

#### 4.2.3. Organic Reactions

Another important application of heterogeneous catalyst supported TiO_2_ derived from the Fisher-Tropsch synthesis, which can convert various carbon sources (coal, natural gas, and biomass) into long chain hydrocarbon via syngas [103]. It is a promising way to produce environmentally benign fuels with no sulfur and nitrogen compounds [116]. In this case, certain transition metals supported with TiO_2_ have frequently applied as catalyst. Among them, Co/TiO_2_ is considered as the preferred catalyst due to its high selectivity for long chain linear paraffin, high resistance toward deactivation by water, and low activity for the competitive water gas shift reaction [25]. Several studies indicated that activity of Co catalyst depended on the number of exposed metal sites [90, 93, 95, 96]. Therefore, the Co/TiO_2_ catalyst system may increase the dispersion of active Co metal species. Meanwhile, TiO_2_ is suitable for the practical application due to the low cost, safety, and the chemical stability. Furthermore, it is reported that the strength of Co support interaction of Co/TiO_2_ was in the middle of those on Co/SiO_2_ and Co/Al_2_O_3_ [99, 100]. It found that the activity of the Co/TiO_2_ catalyst for Fischer-Tropsch synthesis largely depended on the crystal phase of TiO_2_ support, the reduction degree of Co, and the surface area of Co metal. Rutile TiO_2_ gives more optimum reduction degree of Co with almost 60% as compared to the anatase [204, 205].

**Figure 7:**
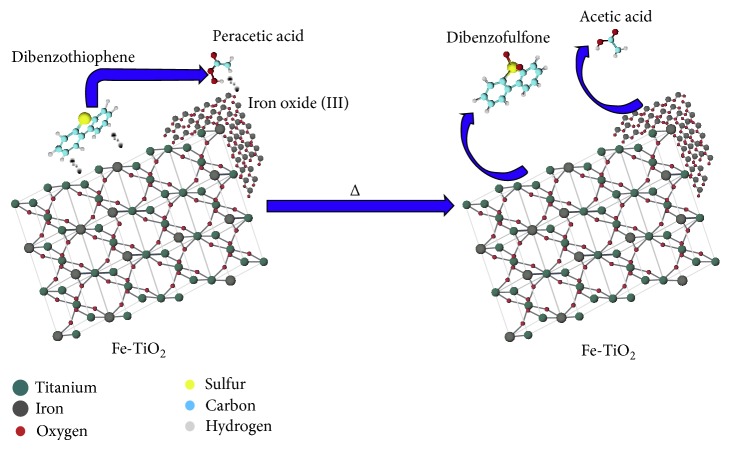
Oxidative removal of dibenzothiophene in a biphasic system using sol-gel Fe/TiO_2_ catalysts and H_2_O_2_ promoted with acetic acid [208].

Another interesting application of TiO_2_ supported catalyst is the use of Fe/TiO_2_ in petroleum refining industries. Several routes can apply the Fe/TiO_2_ catalyst system including oxidation, adsorption, hydrodesulfurization, oxidative desulfurization, and biodesulfurization for organosulfur compounds removal from crude oil and refined petroleum products [2, 59]. This is due to the surface of Fe/TiO_2_ catalyst possess electron-hole pairs and free OH radicals [92]. Highly reactive OH radicals can also be formed by the reaction of the hole with OH–, which are able to generate oxidizing radicals in the presence of peroxides and may perform the oxidative desulfurization better due to recombination reactions occurring via free radical mechanisms [204]. Rather than that, the hydrophilic-hydrophobic character of Fe/TiO_2_ facilitate dibenzothiophene oxidation as a phase transfer catalyst. The coordination of acetic acid and peroxyacetic acid with Fe^3+^ and Ti^4+^ present at the Fe/TiO_2_ surface which in turn perform a superoxides its surface [208] (Figure 7).

### 4.3. Electrochemical Applications

TiO_2_ is an interesting material to evaluate as a heterogeneous catalyst support for electrochemical applications, not only because of its high stability cathode potentials in acidic and hydrous environment [19, 34], but also because of the recent reports introducing Ti-containing material into the electrodes [118, 142]. Substoichiometric Ti_*n*_O_2*n*−2_ has been studied and performs well as a catalyst support in cathodes [90, 126]. It has been reported that once metals are deposited on TiO_2_, it indicates an increased electrochemically active area [139, 142]. The incorporation of TiO_2_ into the cathode indicated the improved methanol tolerance and its proton conductor properties [111].

## 5. Conclusion and Suggestions

TiO_2_ is a reducible metal oxide and strongly reacts with noble metals compared to other metal oxides. For this reason, TiO_2_ has attracted much attention for application as heterogeneous catalyst support in many reactions. It is inferred that catalyst support on TiO_2_ with different structures might exhibit different physicochemical properties and catalytic activities. Generally, pure TiO_2_ possesses abysmal electronic conductivity. It is proposed that substoichiometric TiO_2_ is prepared in order to improve its conductivity. Although the electronic conductivity improved by utilizing TiO_2_ as a heterogeneous catalyst support, the stability was compromised after extensive polarization at high oxygen electrode potentials. Therefore, another method that could improve electronic conductivity is to dope TiO_2_ with* n*-type dopants, including niobium (Nb) or tantalum (Ta). It has been reported in literature that TiO_2_, with a/an rutile/anatase structure doped Nb, had a significantly greater electronic conductivity compared to the native TiO_2_. The presence of Ti^3+^ species was observed, which was induced by the partial replacement of Ti^4+^ by Nb^5+^. Therefore, in all cases, Nb TiO_2_ is both electrochemically and thermally stable, which can further promote explorations for heterogeneous catalyst support.

## Figures and Tables

**Table 1:** List of advantages and disadvantages of heterogeneous catalysis system.

Type of catalysis support	Advantages	Disadvantages
Organic polymer	(i) Easy and versatile functionalization, especially for the polymer containing aryl group (ii) Hydrocarbon polymers are chemically inert-support does not interfere with catalytic groups (iii) It can be prepared with a width range of physical properties (porosity, surface area, and solution characteristics)	(i) It has poor heat transfer ability (ii) It has poor mechanical properties which prevent from the pulverization during stirring process in reactor (iii) Commercial polymers are not always very defined and often contain unknown impurities (iv) Physical properties vary widely depending on molecular weight and chemical nature

Metal	(i) It can induce some catalytic activity as homogeneous catalyst but more selectivity (ii) It is easy to separate from the product (iii) It gives rise to less corrosion (iv) It can be used for long periods without sign of deterioration in properties	(i) Optimization of the reaction condition is more complex because there are more variables (ii) leaching problem brought by the Van de Waals link between the catalyst

Carbon	(i) It has high surface area due to porous structure (ii) It has relatively small amount of chemically bonded heteroatoms (mainly O_2_ and H_2_)	It could not be used for hydrogenation reaction >700 K or in the presence of O_2_ > 500 K because it may become gasified to yield methane and CO_2_, respectively

Dendrimer	(i) It coordinates strongly with metal catalyst (ii) It leads to recyclable catalyst system and does not suffer from mass transfer limitation (iii) It has well-defined macromolecular structure to precisely control catalyst support (iv) Uniform distribution	(i) It suffers from diminished activity due to the reduction in accessibility accessibility (ii) It depends on swelling properties influenced by catalytic performance
